# Risks in the Business of AI

**DOI:** 10.1007/978-3-030-51110-4_6

**Published:** 2020-08-12

**Authors:** Christoph Bartneck, Christoph Lütge, Alan Wagner, Sean Welsh

**Affiliations:** 5 HIT Lab NZ, University of Canterbury, Christchurch, New Zealand; 6 Institute for Ethics in Artificial Intelligence, Technical University of Munich, München, Germany; 7 College of Engineering, Pennsylvania State University, University Park, PA USA; 8 Department of Philosophy, University of Canterbury, Christchurch, New Zealand

## Abstract

This chapter discusses the general risks that businesses face before considering specific ethical risks that companies developing AI systems and robots need to consider. Guidance on how to manage these risks is provided. It is argued that companies should do what is ethically desirable not just the minimum that is legally necessary. Evidence is given that this can be more profitable in the long run.

This chapter discusses the general risks that businesses face before considering specific ethical risks that companies developing AI systems and robots need to consider. Guidance on how to manage these risks is provided. It is argued that companies should do what is ethically desirable not just the minimum that is legally necessary. Evidence is given that this can be more profitable in the long run.

AI is not just a technology that concerns engineers and technical staff. It is a matter of business. Risks associated with advanced technology are likely to increase enormously in the years to come. Doing business with AI comes with many risks. Among the most significant are the risks associated with severe accidents. Take the example of autonomous vehicles, an AV out of control might cause great harm to other people and property. An AI system for controlling the power grid or other infrastructure functions might cause enormous damage if control is lost.

Such risks are not a matter for individuals only. They have consequences for companies, which can be critical and put them out of business entirely. Therefore, many large companies have entire departments for risk management. It is not our purpose here to discuss the more classic risks. Such risks include country risks, political risks, currency risks and related risks. These are well-known and well-understood by companies and insurers. However, the largest multinational global companies, who have the greatest number of skilled personnel and advanced scientific capabilities, are becoming increasingly aware of ethical risks . The more obvious ethical risks are corruption, discrimination or systematic abuse of human rights. The development of advanced technology capable of psychologically influencing people is also a risk. In the globalised world however, such risks might turn into economic risks eventually. There are several mechanisms through which this can occur:

1. **Ethical risk to reputation**
  The reputation of a company may get damaged significantly. This is something not to be underestimated. The brand of large companies is often their most important asset. For example, in 2018, the value of the brand Apple was estimated to be USD 182 billion by Forbes. BMW’s brand was valued at USD 31.4 billion. It would be foolish for such a company to damage their brand for very short-run purposes.
2. **Ethical risk to stock price**
  The stock price of a company might be affected greatly by ethical scandals. BP, for example, suffered great value to its stock price as a result of the Deepwater Horizon disaster in the Gulf of Mexico. In the month following this crisis, its stock price fell from USD 60 to USD 27 in a month. A fall in stock price can be something that destroys careers or leads to takeovers of the entire company. So this is something successful CEOs will seek to avoid.
3. **Ethical risk of legal fines**
  Finally, underestimating ethical risks can lead to significant legal fines. This is something that a number of large companies had to learn the hard way. For example, in the aftermath of the Deepwater Horizon scandal, BP has been forced to pay more than USD 65 billion in damages, fines and clean up costs. Also, Siemens had, before 2006, completely underestimated the issue of systematic corruption within their company. This risk was not regarded as significant, as it did not result in economic problems. However, eventually Siemens had to pay around EUR 2 billion in legal fines, which is a sum that even such a large company would prefer to avoid. As of 2017, according to CNN, the cumulative costs of the Dieselgate scandal for VW are in the order of USD 30 billion.

## General Business Risks

Some of the key risks faced by companies are:

### Functional Risk

 Functional risk is simply the risk of the functionality of the system failing. For example, in any software and hardware system there is always the risk that the system may fail when released to the general public. A component may fail in certain unanticipated situations. Sometimes an update patch for a software system may fail and cause the “breaking” of the system itself or another system it interacts with it. Updates often break integration between systems provided by different vendors.

### Systemic Risk

 Systemic risks are risks that affect a whole system. Systemic risk in finance, for example, is the risk of the entire financial system collapsing. The Global Financial Crisis of 2007–08 was caused by widespread loan defaults in the US subprime market. Subprime loans are given to people with weak credit ratings. Defaults stressed the major US mortgage lenders Fannie Mae and Freddie Mac and many home owners abandoned their houses. This led to a collapse in confidence that exposed inadequate risk models. One of the causes of the collapse of Lehman Brothers was wholesale underpricing of the risk of mortgage defaults and lowering real estate prices. This was due to faulty financial models. When Lehman Brothers filed for bankruptcy in 2008, banks stopped trusting each other and started to refuse to lend to each other. The global financial system required massive bailouts from governments.

Sophisticated AI systems are widely used in finance. Indeed automated trading systems are widely used by investment bankers and financial traders. The Flash Crash of 2010 which lasted for 36 min caused the US stock market to lose 9% of its value. High-frequency traders that used AI systems were involved in this incident. There are fears that use of such systems might “blow up” the market and cause a recession or even a depression.

### Risk of Fraud

Computer systems have been used to perpetrate fraud . One of the largest was the Dieselgate scandal. Volkswagen deliberately designed their emissions reduction system to only function during laboratory tests. As a result of this criminal deception their cars passed tests in labs but emitted up to forty times these volumes on the road. Even so, Volkswagen promoted their fraudulently obtained “green” credentials. The software used to detect the laboratory test was relatively simple and used parameters, such as the steering wheel inclination (Contag et al. 2017). Still, this is an example of how a computer system can be used in large scale frauds.

### Safety Risk

Robots that control industrial production present physical risks to the people that work around them. People may be harmed by force of collisions or harmed by the objects that a robot carries or moves. The Deepwater Horizon explosion is an example of a situation where a system failure led to a catastrophe that killed eleven workers. Similarly, autonomous vehicles have, on occasion, caused accidents that have led to fatalities. Uber and Tesla AVs have been involved in fatal collisions.

## Ethical Risks of AI

There are ethical risks for AI and robotic technologies. Managing them becomes a crucial task for managers in the globalised economy. With this in mind what are the risks of AI technologies and robotics? Some of the main ethical risks are.

### Reputational Risk

Systems that appear  biased or prejudiced can cause great reputational damage. Hot topics in this area include facial recognition and loan approval systems. However a spirited campaign of “testing, naming and shaming” has dramatically increased the ability of commercial systems to correctly recognise the faces of females and minorities (Buolamwini and Raji 2019).

One of the first major scandals in the still young social media economy is the Cambridge Analytica scandal, which greatly damaged the reputation of Facebook. Facebook supplied data obtained by Cambridge Analytica and which was used for targeted political advertising.

### Legal Risk

Legal risk encompasses  situations where a system becomes too successful and is viewed as causing an anti-competitive environment. This situation may attract the attention of governmental regulators capable of levying large fines. In 2004, Microsoft, for example, was fined EUR 497 million by the European Union for anti-competitive behaviour. In 2018, the European Commission imposed a record fine of USD 5 billion on Google for antitrust violations of the Android technology.

### Environmental Risk

Similarly system failures can  cause environmental disasters. The Bhopal disaster in India, for example, was the result of a gas leak at an Union Carbide factory in Bhopal India. The leak resulted in an explosion and release of methyl isocyanate. The immediate explosion caused the death of nearly 4,000 people. The gas release however may have harmed more than half a million people. Several thousand would eventually be determined to have suffered permanent damage from exposure to the toxic substance. Union Carbide eventually paid approximately USD 470 million in fines and restitution.

### Social Risk

Social risk includes actions  that may include the people, society or communities around the business. These risks may include increased traffic, noise pollution, and issues related to worker morale. Social risks related to AI may include technology induced increased social isolation, increased inequality, and local community issues surrounding the acceptable uses of technology and AI. For example, the use of Google goggles generated numerous issues surrounding the privacy and the use of an integrated camera in private places.

The examples above briefly introduce some of the more ethically charged risks that companies must manage to survive in the modern world. There are numerous other risks besides these (currency risk, commercial risk etc.) which we do not cover here.

## Managing Risk of AI

One important question is  how these risks can be assessed, and even quantified, if possible. Usually, it makes a big difference especially to large companies, if a risk can be estimated in money. We have already mentioned the loss in the value of a company brand, in stock price and in legal fines, but there are also other attempts in quantifying reputational risk, by a mix of asking external experts, doing surveys with employees or other measurements. Again, this leads to a value which will then have consequences for a company, internally and externally.

For AI companies as well as in others, there are several ways of dealing with these risks. First there are legal methods of establishing or improving existing safety regulations. For example, in the case of autonomous cars, laws are being adjusted to accommodate the inclusion of these technologies. At the same time, industry best practices are also being developed and implemented.

Second, regulations surround ancillary industries may need to be developed. For autonomous cars, this includes changes to insurance regulations, perhaps including the introduction or updating of compulsory insurance to meet the needs of this rapidly developing technological.

Finally, many companies will certainly, voluntarily or not, be compelled to go beyond the minimum legal standards for reasons of their own ethical values and addressing risks. Being a good corporate citizen implies controlling risks—in your own company as well as in the supply chain. For example, when the Rana Plaza building in Bangladesh collapsed in 2013, the public and many consumers viewed this as a problem of large multinational companies in the clothing industry and not just as a local problem. Similar viewpoints have been held in the case of Apple or Samsung as being responsible for child labour in the cobalt mines used in their supply chain.

DuckDuckGo, a search engine competing with Google, heavily advertises the fact that is does not collect data from people. As the saying goes in AI, “if you are not paying, you’re the product.” Personal data has considerable value to those seeking to target their marketing. DuckDuckGo is seeking to differentiate themselves from Google in the search engine market by not collecting user data. Whether it can displace Google from its dominant position in search and targeted advertising marketing by doing this remains to be seen. The value placed on personal data for the purposes of AI has been discussed in Sect. 10.1007/978-3-030-51110-4_8.

## Business Ethics for AI Companies

Business ethics in a global  environment has, to a significant part, become an instance of risk management for AI companies. Mechanisms like reputation, stock prices and legal fines exhibit a business case for ethics and may help to convince companies to be active in this area.

Let’s consider another example: The Malampaya project was a pipeline project by the Shell corporation in the Philippines in 1998 (Pies 2010). There were three possible ways to build this pipeline. First, it could have been built primarily on land, which would have been the cheapest way, but one which would have put the biodiversity of the region at great risk.

The second option would have been to build the pipeline in a direct, more expensive underwater route, but that route would have gone through a territory that was considered holy by indigenous people (who, however, had no legal claim to it).

Still, Shell eventually decided to go for a third, and much more costly option: to build the pipeline underwater, but on a different and much longer route around an island, which led it far away from the holy territory. There was no legal obligation for Shell to do so, and the additional cost was around 30 million US dollars. Although, this amount is not a significant cost for a company like Shell, it still represents a cost that would have been avoided if possible.

The deciding factor at the time probably was the aftermath of the 1995 Brent Spar Case: Shell tried to sink an unused oil platform in the North Sea, resulting in protests from Greenpeace and others and even in an initial boycott by consumers. Eventually, Shell abandoned their plan (though Greenpeace was later forced to admit that there was much less residual oil in the platform than it had alleged). Shell may have been trying to avoid a similar scandal.

What is interesting, however, is that in 2007 independent calculations by the World Resources Institute came to the conclusion that what seemed at first to be the most expensive option actually turned out to have been the more economical one, when taking into account other costs such as the costs of delayed completion. The indigenous people in question, while not having been able to stop the project entirely, still could have significantly delayed it by going to court. The delays would have resulted in penalties and other costs. Therefore, the overall calculation of all these resulted in the chosen option actually being proven to have made economic sense.

Not all cases have happy endings. Still, it is valuable for companies to consider examples such as this. These case studies may encourage companies to include ethics and environmental information in their calculates and to invest money in Corporate Social Responsibility initiatives. The same might hold in the area of AI, these companies should improve their inclusion of ethics related considerations, calculating over the long run, and consider systematically the interests of others who may be relevant for their future sustainable operations.

From a more general point of view, taking on ethical responsibilities (without waiting for changes in the legal framework) has become both a necessity as well as a competitive advantage for companies. Studies show that it helps raise profits (Wang et al. 2016), secure the capability for innovation, improve market position (Martinez-Conesa et al. 2017), improve risk management (Shiu and Yang 2017) and customer satisfaction (Lins et al. 2017) and also motivate employees and recruit new talent (Saeidi et al. 2015).

One word of caution seems appropriate: Companies can do a lot of things on their own, but they cannot do everything. In some sectors of the industry, it will be more difficult for a company to take responsibility on their own. Consider the banking sector. Broadly speaking, there is not as much room for direct action by banks as in other industries, simply because the banking sector is very tightly regulated.

The situation in AI could become similar. As a product developed by AI may be capable of making important decisions in the world and as it is difficult or impossible to argue or appeal to these programs or algorithms, we will need specific ethical guidelines for AI. This does not necessarily mean detailed regulation. But rather guidelines that are the subject and focus of societal discussion. Hence, they need to address important problems that people care about—even if they might not be able to solve each of these problems.

## Risks of AI to Workers

Many writers have predicted the emergence of “mass technological unemployment” resulting from the increasing use of AI and robots. The pessimistic line is that humans will all eventually be made redundant by machines. Numerous reports have made alarming predictions that huge numbers of human jobs will be automated away (Frey and Osborne 2017). For example, there are around three million truck drivers in the United States. If a viable autonomous truck were available tomorrow, in theory, all three million truck drivers could be made redundant very quickly. There are many stories about how AI and robotic will make everyone redundant. However, these stories are based on uncritically accepted assumptions.

For example, Frey and Osborne’s projections have been widely criticised. They predicted that nearly half the jobs in the workforce were vulnerable to automation over the next 20–30 years. However, an OECD report using different assumptions arrived at a much less alarming figure of 9% (Arntz et al. 2016).

Certainly, if the predictions of looming “mass technological unemployment” were true, we would expect to see lengthening unemployment lines in the most advanced technological nations. However, prior to the COVID-19 pandemic, employment in the United States is currently at 30 year lows. So, it would seem that new jobs are being created to replace those lost to automation.

**Fig. 6.1:**
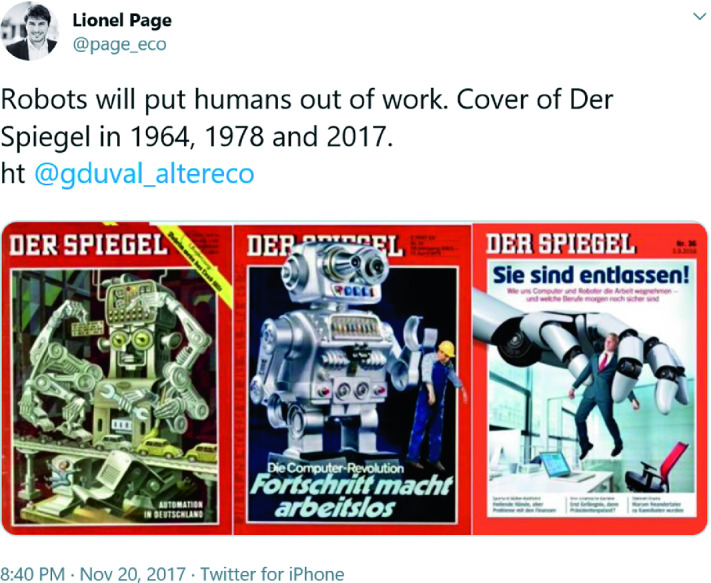
Der Spiegel covers on mass unemployment

Another myth-busting consideration is that headlines predicting the replacement of workers by robots are not new. The German current affairs magazine *Der Spiegel* has run three cover stories predicting that robots would put people out of work, in 2017, 1978 and 1964 as shown in Fig. 6.1.

The main counter-argument to the claim that AIs and robots will put us all out of work is the fact that automation has been going on since the Industrial Revolution. Technological advancement does disrupt employment. This is undeniable. However, new technology means workers can do more with less and they can do more interesting and valuable things as wealth in a society increases. Historically, new jobs have been created to replace the old ones. If the predictions of mass technological unemployment were true, then we would expect to see high unemployment in technologically advanced states. There is no sign of this happening yet.

Discussion Questions:

- Are you worried about AIs and robots putting you out of work or excited by the possibilities of new technology? Explain.
- Give some examples of how bias could be introduced into an AI.
- Do you think there should be a compulsory insurance scheme for the risks associated with AVs?

Further Reading:

- Eric Bonabeau. Understanding and managing complexity risk. *MIT Sloan Management Review*, 48(4):62, 2007. URL https://sloanreview.mit.edu/article/understanding-and-managing-complexity-risk/
- A Crane and D. Matten. *Business Ethics. Managing Corporate Citizenship and Sustainability in the age of Globalization*. Oxford University Press, 2007. ISBN 978-0199697311. URL http://www.worldcat.org/oclc/982687792
- Christoph Luetge, Eberhard Schnebel, and Nadine Westphal. Risk management and business ethics: Integrating the human factor. In Claudia Klüppelberg, Daniel Straub, and IsabellWelpe, editors, *Risk: A Multidisciplinary Introduction*, pages 37–61. Springer, 2014. Doi: 10.1007/978-3-319-04486-6. URL https://doi.org/10.1007/978-3-319-04486-6.

